# Dentin pretreatment and adhesive temperature as affecting factors on bond strength of a universal adhesive system

**DOI:** 10.1590/1678-7757-2016-0500

**Published:** 2017

**Authors:** Bruna Gabrielle da Silva Sutil, Alexandre Henrique Susin

**Affiliations:** 1 Universidade Federal de Santa Maria, Programa de Pós-Graduação em Ciências Odontológicas, Santa Maria, RS, Brasil; 2 Universidade Federal de Santa Maria, Departamento de Odontologia Restauradora, Santa Maria, RS, Brasil

**Keywords:** Adhesives, Dentin, Temperature, Dental air abrasion, Bonding

## Abstract

**Objectives:**

To evaluate the effects of dentin pretreatment and temperature on the bond strength of a universal adhesive system to dentin.

**Material and Methods:**

Ninety-six extracted non-carious human third molars were randomly divided into 12 groups (n=8) according to Scotchbond Universal Adhesive (SbU) applied in self-etch (SE) and etch-and-rinse (ER) mode, adhesive temperature (20°C or 37°C) and sodium bicarbonate or aluminum oxide air abrasion. After composite build up, bonded sticks with cross-sectional area of 1 mm^2^ were obtained to evaluate the microtensile bond strength (μTBS). The specimens were tested at a crosshead speed of 0.5 mm/min on a testing machine until failure. Fractured specimens were analyzed under stereomicroscope to determine the failure patterns in adhesive, cohesive (dentin or resin) and mixed fractures. The microtensile bond strength data was analyzed using two-way ANOVA and Tukey's test (α=5%).

**Results:**

Interaction between treatment and temperature was statistically significant for SbU applied in self-etch technique. Both dentin treatments showed higher bond strength for ER mode, regardless of adhesive temperature. When compared to control group, sodium bicarbonate increased bond strength of SbU in SE technique. Adhesive temperature did not significantly affect the μTBS of tested groups. Predominantly, adhesive failure was observed for all groups.

**Conclusions:**

Dentin surface treatment with sodium bicarbonate air abrasion improves bond strength of SbU, irrespective of adhesive application mode, which makes this approach an alternative to increase adhesive performance of Scotchbond Universal Adhesive to dentin.

## Introduction

With the development and improvement of aesthetic restorative materials, adhesive systems have become essential elements in various clinical applications. Adhesive systems are responsible for the bonding of restorative material to dental structures. Thus, the longevity of adhesive restoration is directly associated with the effectiveness of adhesive systems^5^.

The adhesion of composite resins to the dentin substrate is based on smear layer treatment^5^. While some adhesive systems require the conditioning of dentin with phosphoric acid, as etch-and-rinse systems (ER), others preserve the smear layer by incorporating it into the adhesive layer, they are the self-etch systems (SE). Self-etch systems use an acidic primer, which can be separated from the adhesive or can be all-in-one, in which the primer and adhesive are present in a single bottle^5^. New universal or multi-mode adhesives are single-step self-etch adhesives that can be applied both to the ER and SE techniques^12^ ^,^ ^21^.

The ER technique is more sensitive, as it is susceptible to operator error and can affect the condition of the substrate, since this technique can lead to the collapse of the collagen network due to the rinsing-drying steps. In addition, incomplete resin infiltration of demineralized dentin can occur^17^. Thus, self-etching primer systems have been developed to minimize the sensitivity of the technique and reduce the time of clinical application^8^ ^,^ ^17^. However, some failures of restoration using this type of adhesive can be associated with the inability of the adhesive to penetrate correctly into the smear layer and reach the underlying dentin^27^ ^,^ ^30^.

Dentin adhesion depends not only on the successful permeation of the adhesive into the dental substrate, but also on the mechanical properties and quality of the polymer formed. Thus, the solvents and the water present in the adhesive should ideally be removed to a sufficient degree because residual solvents can inhibit the polymerization and negatively affect the mechanical properties of the adhesive^16^ ^,^ ^26^. Hence, a clinical approach to improve the adhesive properties indicates the use of warm air^9^ ^,^ ^13^ ^,^ ^16^ ^,^ ^19^ ^,^ ^20^ ^,^ ^26^ to facilitate the evaporation of the solvent as well as the use of preheated adhesive systems^11^ ^,^ ^18^ ^,^ ^25^ ^,^ ^28^ in order to increase the speed of permeation of the monomers and the evaporation of the residual monomeric content. The quality and the stability of the hybrid layer can be influenced by the heating of adhesive through the stimulation of their chemical reactions and, thus, some properties of the monomeric solutions, such as degree of monomer conversion and viscosity, can be changed by temperature^1^ ^,^ ^11^ ^,^ ^18^ ^,^ ^23^ ^,^ ^28^. Temperature can increase the mobility of free radicals, benefiting the polymerization of the adhesive by increasing the degree of conversion^1^, besides decreasing the viscosity and improving the speed of spreading of the adhesive and deeper penetration into dentin^11^ ^,^ ^18^ ^,^ ^25^ ^,^ ^28^.

Although new adhesive systems should be developed in order to improve performance, we can use different dentin clinical manipulation techniques to increase the bonding and, consequently, long-term clinical success. Alternatively, the use of some dentin pretreatments such air abrasion techniques may improve the bonding strength, through mechanical modification of the dentin substrate^8^.

Abrasion with aluminum oxide particles has been used to increase the bonding of metallic surfaces to resinous materials, to prepare and clean the cavity, and to remove decayed tissue and faulty restorations^6^ ^,^ ^24^. Moreover, abrasion with sodium bicarbonate has been used for prophylaxis of the dentinal surface and removal of plaque and debris formed during cavity preparation. Sodium bicarbonate abrasion is superior to other cleaning methods, such as the rubber cups, mainly in areas that are difficult to access, such as during deeper cavity preparations^3^ ^,^ ^22^. However, air abrasion has also been used in dentin pretreatment, because it increases the surface roughness and the area available for adhesion and thus improves the interfacial contact between the dentin and the adhesive^2^ ^,^ ^6^ ^,^ ^8^. Furthermore, removal of the smear layer by abrasion with aluminum oxide particles can improve adhesive infiltration into dentin, increasing bond strength^5^.

Thus, the aim of this *in vitro* study was to evaluate the effect of pretreatment of the dentin surface on the bond strength of a universal adhesive system to dentin and the impact of temperature on adhesion. The tested hypotheses were: 1) The surface pretreatments increase the bond strength in both adhesive techniques; 2) Pre-heating of the adhesive system improves the adhesion.

## Material and methods

### Selection and preparation of teeth

**Figure 1:**
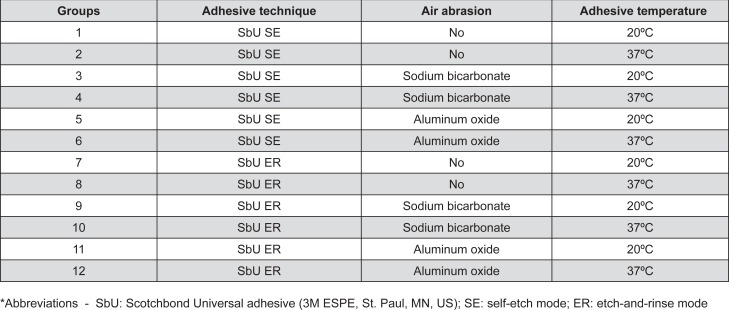
Experimental groups

**Figure 2:**
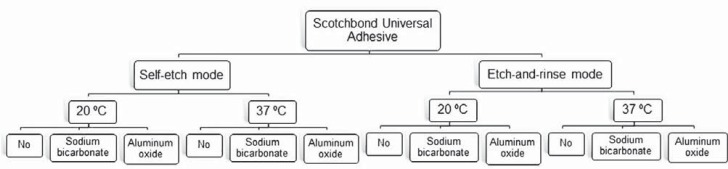
Experimental groups

This study was previously submitted to the Institutional Research Ethics Committee (47522515.7.0000.5346). Ninety-six intact human third molars (n=8) were used. Sample size was calculated according to the expected means, 80% statistical power and 5% significance level, using the OriginPro 2015 software (OriginLab Co; Northampton, MA, USA). They were stored in a chloramine T 0.5% solution at a temperature of 4°C for one month. The teeth were then removed from the disinfectant solution, washed abundantly, stored in distilled water of the same temperature, and used within six months. The root portion of the teeth was removed and the occlusal third was sectioned with a diamond disk at low speed and cooled with water, using a Labcut^®^ 1010 cutting machine (Extec; Enfield, CT, USA). The sectioned root portions were then embedded in PVC tubes (Tigre S.A.; Joinville, SC, Brazil) with New wax sticky wax (Technew; São Paulo, SP, Brazil). The enamel surfaces were abraded with #180 grit SiC paper under running water until exposure of a flat dentin surface. To standardize the smear layer, dentinal surfaces were polished under running water with #600 grit SiC paper for 60 seconds using an Arotec PL 4 polishing machine (Arotec; São Paulo, SP, Brazil). The teeth were randomly divided into 12 experimental groups by *www.random.org* site (Randomness and Integrity Services Ltd; Dublin, Ireland) according to the adhesive system method used (self-etch or etch-and-rinse techniques), surface treatments (sodium bicarbonate or aluminum oxide air abrasion) and temperature of the adhesive (20°C or 37°C) (Figures 1 and 2).

### Abrasive and restorative procedures

In the groups that received surface treatments, the dentin was air-abraded with Laxis Sonic BP (Schuster; São Paulo, SP, Brazil) sodium bicarbonate jet (15-300 um) for 15 seconds, at a distance of 5 mm, a pressure of 60 psi, and an angle of 90° between the jet and dentin. The dentinal surface received an air/water spray jet for 30 seconds and was dried with absorbent paper. In another treatment, the dentinal surface was abraded with aluminum oxide particles (50 μm) with an angle of 90° between the jet and dentin, for 10 seconds, at a distance of 5 mm and a pressure of 60 psi, using a Micro-jato jet (Bio Art; São Carlos, SP, Brazil). The dentinal surface was then washed with an air/water spray for 15 seconds and dried with absorbent paper.

**Figure 3:**
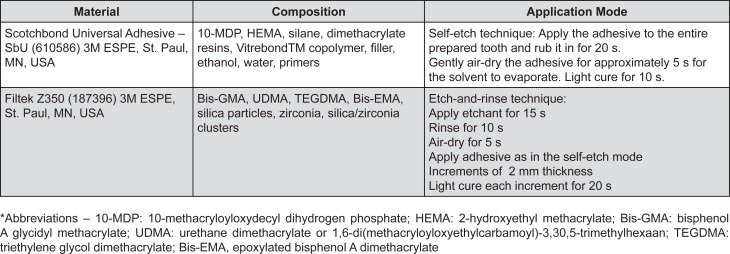
Material (batch number), composition and application mode according to the manufacturer's instructions

The Scotchbond Universal (SbU) adhesive (3M ESPE; St Paul, MN, USA) was used in etch-and-rinse (ER) and self-etch (SE) techniques at a room temperature of 20°C or heated in the specific device to 37°C. Application mode was performed according to the manufacturer's instructions (Figure 3). After the adhesive procedures, a composite resin restoration was built on the dentinal surface at increments of 2 mm in thickness, using the nanofilled dental resin Z350 (3M ESPE; St Paul, MN, USA) in the shade A2. The resin layers were photocured individually for 20 seconds using RADII (SDI; São Paulo, SP, Brazil) with an intensity of 800 mW/cm^2^. Thereafter, the specimens were stored in distilled water for 24 hours in order to be prepared for the microtensile bond strength test.

### Microtensile bond strength test (μTBS)

A diamond disk was used at low speed and under refrigeration in order to prepare the specimens for the microtensile test. These specimens were mounted on a Labcut^®^ 1010 cutting machine (Extec; Enfield, CT, USA). The teeth were sectioned into slices parallel to the long axis and then cut again perpendicular to the first sections. This was done in order to obtain specimens in the form of sticks with an adhesive area of 1 mm^2^, measured through a digital pachymeter (Kingtools, São Paulo, SP, Brazil). The specimens were examined through a Discovery V20 stereomicroscope (Carl-Zeiss; Oberkochen, Germany), with an amplification of 10x. Thus, sticks with bubble inclusions and adhesive failures were excluded. Each specimen was attached to a μTBS testing device with Super Bonder cyanoacrylate-based adhesive gel (Loctite Ltda; São Paulo, SP, Brazil) and subjected to a tensile force in a EMIC DL-2000 universal test machine (EMIC; São José dos Pinhais, PR, Brazil), with a 50 KgF load cell at a speed of 0.5 mm/min.

### Analysis of failure mode

To determine if the failure that occurred was adhesive (fracture at the interface between the resin and dentin), cohesive (fracture within the body of the resin or dentin), or mixed (adhesive fracture combined with cohesive fracture), the specimens were analyzed by Discovery V20 stereomicroscopy (Carl-Zeiss; Oberkochen, Germany) with 40x amplification.

### Statistical analysis

The Shapiro-Wilk test was employed to verify a normal distribution. Bond strength data were analyzed separately using two-way ANOVA (air abrasion *vs*. temperature) for etch-and-rinse and self-etch strategies. The Tukey test was used for multiple comparisons, with a significance level of 5%. All statistical analyses were performed in the OriginPro 2015 software (OriginLab Co; Northampton, MA, USA).

## Results

Means and standard deviations of the adhesive techniques tested are presented according to surface treatment and temperature in Table 1. A two-way ANOVA test showed that the factor temperature (p=0.689) was not statistically significant for SbU in SE mode, while the dentinal treatment (p<0.001) and the interaction of factors (p=0.012) had significant effect on the SE technique. For SbU in ER mode, temperature (p=0.002) and treatment (p<0.001) were significant.

**Table 1:** Microtensile Bond Strength (MPa) values (means and standard deviations) of the different experimental groups (*)

Surface treatment	SbU Self-etch		SbU Etch-and-rinse	
	20°C	37°C	20°C	37°C
No (control)	36.14 (6.63)^A^	32.92 (6.19)^A^	30.10 (5.93)^a^	33.78 (4.08)^a^
Sodium bicarbonate	65.45 (4.46)^B^	59.53 (11.48)^B^	49.65 (2.38)^b,c^	51.44 (7.62)^c,d^
Aluminum oxide	37.46 (13.42)^A^	49.67 (7.2)^C^	44.26 (8.62)^b^	55.08 (3.74)^d^

(*) Similar capital letters (self-etch) and lowercase letters (etch-and-rinse) are not statistically significant (p<0.05)

Both surface pretreatments showed higher values of microtensile bond strength when the SbU adhesive was used in the ER technique, regardless of the temperature of the adhesive. For the application in SE mode only, sodium bicarbonate air abrasion increased the bond strength when compared to control group.

**Figure 4:**
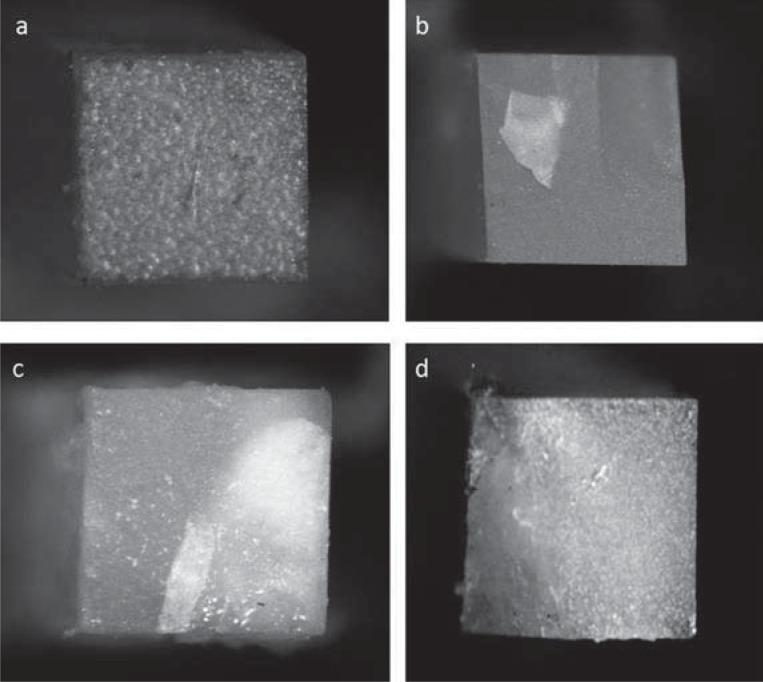
Images representing the different fracture patterns: a-) Image representing adhesive fracture (40x magnification); b-) Image representing cohesive fracture in resin (40x magnification); c-) Image representing cohesive fracture in dentin (40x magnification); d-) Image representing mixed fracture (40x magnification)

Analysis of the fracture pattern presented predominantly adhesive failures for all groups (Figure 4a). However, the four types of failures were observed in all groups (Figure 4). Descriptive results are presented in Table 2.

**Table 2:** Number and percentage of specimens (%) according to the fracture pattern mode

Application mode	Surface treatment - adhesive temperature		Fracture pattern			
			A	Cr	Cd	M
Self-etch	Control -	20°C	90 (84.11)	12 (11.21)	3 (2.81)	2 (1.87)
	Control -	37°C	97 (85.09)	11 (9.65)	4 (3.51)	2 (1.75)
	Sodium bicarbonate -	20°C	77 (60.16)	36 (28.12)	9 (7.03)	6 (4.69)
	Sodium bicarbonate -	37°C	70 (73.68)	19 (20.00)	4 (4.21)	2 (2.11)
	Aluminum oxide -	20°C	75 (70.75)	23 (21.70)	2 (1.89)	6 (5.66)
	Aluminum oxide -	37°C	75 (86.21)	8 (9.19)	3 (3.45)	1 (1.15)
Etch-and-rinse	Control -	20°C	87 (83.65)	8 (7.69)	5 (4.81)	4 (3.85)
	Control -	37°C	92 (83.64)	10 (9.09)	5 (4.54)	3 (2.73)
	Sodium bicarbonate -	20°C	94 (73.44)	22 (17.18)	6 (4.69)	6 (4.69)
	Sodium bicarbonate -	37°C	100 (87.72)	9 (7.89)	4 (3.51)	1 (0.88)
	Aluminum oxide -	20°C	87 (72.11)	13 (10.92)	16 (13.45)	3 (2.52)
	Aluminum oxide -	37°C	95 (66.90)	31 (21.83)	12 (8.45)	4 (2.82)

*Abbreviations - A: adhesive fracture mode; Cr: cohesive in resin fracture mode; Cd: cohesive in dentin fracture mode; M: mixed fracture

## Discussion

Universal dental adhesives were developed mainly with regard to dentin; with the aim of simplifying the clinical steps and reducing the sensitivity of clinical techniques. Due to their versatility, they may be used with the etch-and-rinse and self-etch techniques^10^ ^,^ ^29^. As they are a class of recent and little studied adhesives, alternative approaches, such as dentinal pre-treatment^5^ ^,^ ^8^ and pre-heating of the adhesive^11^ ^,^ ^18^ can be carried out to improve adhesive properties.

Cleaning of the dentinal surface with sodium bicarbonate air-powder polishing before adhesive procedures is a very common technique and aims to remove plaque and debris present in the cavity, which may influence bond strength^3^ ^,^ ^15^. In this study, the treatment of dentin with sodium bicarbonate significantly increased the bond strength of the two techniques when compared to control group. This finding contradicts the results of Frankenberger, et al.^7^ (2007), who reported that sodium bicarbonate air-polishing considerably decreased the bond strength, regardless of the adhesive and adhesive technique. Other studies have also demonstrated that adhesion is affected when sodium bicarbonate was applied to the dentin^15^ and the enamel^3^. On the other hand, Rosin, et al.^22^ (2005) found no significant difference in bond strength of dentin when it was abraded with sodium bicarbonate, both for self-etch and etch-and-rinse modes. These findings differ from ours, but it should be taken into account that we used a different adhesive to evaluate the bonding of dentin treated with sodium bicarbonate.

In the present study, the high bond strength values obtained with dentin abraded with sodium bicarbonate may be explained by the modification of the abrasion technique used, as well as by the type of adhesive applied. Regarding the technique, the dentin was rinsed with an air/water jet for twice the time of abrasion with sodium bicarbonate. This step could have removed the sodium bicarbonate particles more effectively. These particles can act as a contaminant and hinder the close contact between the adhesive and the dentin, resulting in the reduction of bond strength that was observed in previous studies^7^ ^,^ ^15^. Moreover, the Scotchbond Universal adhesive system contains the functional monomer 10-methacryloyloxydecyl dihydrogen phosphate (MDP) which has higher bond strength than some universal adhesives^14^ ^,^ ^29^. The MDP has been shown to be effective due to the low solubility of the calcium salt that forms on the hydroxyapatite surface; thus universal adhesive systems are able to establish a chemical bond between specific carboxylic or phosphate groups of functional resin monomers (MDP) and residual hydroxyapatite crystals on the dentin collagen scaffold, enabling a stable bond to dentin ^12^ ^,^ ^14^ ^,^ ^29^.

The pretreatment of dentin with aluminum oxide air abrasion improves the dentinal bond strength due to increased surface roughness and contact between the dentin and the adhesive^2^ ^,^ ^6^ ^,^ ^8^. In addition, the superficial removal of the smear layer by air abrasion could increase the infiltration of the resin monomers into the dentin and thus increase adhesion^5^. In this study, when the adhesive was used in the etch-and-rinse mode, bond strength increased significantly when the dentin was abraded with aluminum oxide. On the other hand, there were no differences in bond strength when the SbU was used in self-etch mode. In partial agreement with these results, previous studies have shown that the abrasion of dentin with aluminum oxide does not interfere with bond strength both in self-etch and etch-and-rinse modes^2^ ^,^ ^5^ ^,^ ^6^ ^,^ ^8^ ^,^ ^27^. The increase in bond strength observed for the SbU in etch-and-rinse strategy may be due to a change in the dentinal surface energy caused by abrasion with aluminum oxide that promoted better interactions between forces of cohesion and adhesion which determine whether wetting (the spreading of a liquid over a surface) occurs, and increasing area available for adhesion^4^ ^,^ ^8^. In addition, acid conditioning removes the smear layer and can remove aluminum oxide particles left on the dentinal surface, thus exposing the dentinal tubules, improving the infiltration of the adhesive into the dentin and enhancing resin tag formation^5^ ^,^ ^6^ ^,^ ^8^. Therefore, our first hypothesis was partially accepted.

Temperature influences some properties of monomers by decreasing the viscosity and by increasing the spreading speed of the adhesive and its deeper penetration into dentin, besides increasing the degree of conversion of monomers, which influences the adhesive effectiveness^11^ ^,^ ^18^ ^,^ ^25^ ^,^ ^28^. In this study, however, pre-heating of the adhesive did not significantly alter the bond strength, except when associated with aluminum oxide groups; thus, the second hypothesis tested was partially accepted. This finding corroborates some studies, which concluded that increase in temperature had no significant effect on an adhesive based on ethanol/water, similar to the SbU tested in this study^11^ ^,^ ^13^. Other studies showed that higher immediate bond strength was associated with the highest temperatures for enamel (40°C)^1^ and dentin (50°C)^18^; however, the adhesive systems that presented these results are classified as etch-and-rinse adhesives, thus being different from the SbU. Another study showed that the heated adhesive (40°C) increased the bond strength for the etch-and-rinse Adper Single Bond adhesive, but did not influence the Clearfil SE Bond, considered a self-etching adhesive^25^.

The stereomicroscopy analysis revealed a predominance of adhesive failures for all experimental groups. This finding corroborates some studies, which showed an adhesive fracture pattern^11^ ^,^ ^12^ ^,^ ^29^. However, other studies showed a pattern of different fractures, predominantly cohesive^6^ and mixed failures^22^. While adhesive failures microscopically represent a rupture in the interface between the resin and dentin characterized by an opening of the dentinal tubules, cohesive failures indicate that the hybrid layer is intact^6^.

Within the limitations of an *in vitro* study, our findings suggest that the dentinal surface can undergo alternative approaches prior to adhesive procedures, for example, pretreatment with sodium bicarbonate air abrasion, in order to improve the performance of the Scotchbond Universal adhesive system. More studies are needed to evaluate the influence of these approaches on the adhesive effectiveness of universal adhesive systems in the short and long term.

## Conclusion

Pretreatment of dentin with sodium bicarbonate air abrasion increases the bond strength of universal adhesive, regardless of whether the etch-and-rinse or the self-etch technique is used. Treatment of dentin using abrasion with aluminum oxide particles influences adhesion only when using the etch-and-rinse application mode. Pre-heating of the adhesive does not significantly influence bond strength.
